# Characterisation of motor cortex organisation in patients with different presentations of persistent low back pain

**DOI:** 10.1111/ejn.15511

**Published:** 2021-11-11

**Authors:** Edith Elgueta‐Cancino, Liba Sheeran, Sauro Salomoni, Leanne Hall, Paul W. Hodges

**Affiliations:** ^1^ Centre of Clinical Research Excellence in Spinal Pain, Injury and Health, School of Health and Rehabilitation Sciences The University of Queensland Brisbane Queensland Australia; ^2^ School of Sport, Exercise and Rehabilitation Sciences University of Birmingham Birmingham UK; ^3^ Biomechanics and Bioengineering Research Centre Versus Arthritis Cardiff University Cardiff UK; ^4^ School of Healthcare Sciences Cardiff University Cardiff UK

**Keywords:** central sensitisation, low back pain, motor control, nociceptive pain, nociplastic pain, transcranial magnetic stimulation

## Abstract

Persistence of low back pain is thought to be associated with different underlying pain mechanisms, including ongoing nociceptive input and central sensitisation. We hypothesised that primary motor cortex (M1) representations of back muscles (a measure of motor system adaptation) would differ between pain mechanisms, with more consistent observations in individuals presumed to have an ongoing contribution of nociceptive input consistently related to movement/posture. We tested 28 participants with low back pain sub‐grouped by the presumed underlying pain mechanisms: nociceptive pain, nociplastic pain and a mixed group with features consistent with both. Transcranial magnetic stimulation was used to study M1 organisation of back muscles. M1 maps of multifidus (deep and superficial) and longissimus erector spinae were recorded with fine‐wire electromyography and thoracic erector spinae with surface electromyography. The nociplastic pain group had greater variability in M1 map location (centre of gravity) than other groups (*p* < .01), which may suggest less consistency, and perhaps relevance, of motor cortex adaptation for that group. The mixed group had greater overlap of M1 representations between deep/superficial muscles than nociceptive pain (deep multifidus/longissimus: *p* = .001, deep multifidus/thoracic erector spinae: *p* = .008) and nociplastic pain (deep multifidus/longissimus: *p* = .02, deep multifidus/thoracic erector spinae: *p* = .02) groups. This study provides preliminary evidence of differences in M1 organisation in subgroups of low back pain classified by likely underlying pain mechanisms. Despite the sample size, differences in cortical re‐organisation between subgroups were detected. Differences in M1 organisation in subgroups of low back pain supports tailoring of treatment based on pain mechanism and motor adaptation.

## Graphical abstract

Features of M1 organisation differed in subgroups of LBP based on presumed mechanisms from pain‐free individuals. Greatest differences were observed in individuals from the mixed group MP of likely contribution of nociceptive input and altered psychosocial features. The cortical drive to back muscles might be adapted to a pattern of protection that is enhanced by fear. This suggests tailoring of interventions for pain mechanism, psychosocial features and motor control issues could be considered.

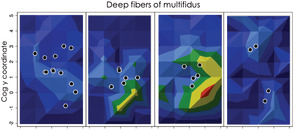

**Abbreviation list**
AEP: active extension pattern
ANOVA: analysis of variance
CoG: centre of gravity
CSQ: Coping Strategies Questionnaire
DM: deep fibres of the multifidus muscle
EMG: electromyography
F: female
FP: flexion pattern
fMRI: functional magnetic resonance imaging
LBP: low back pain
LES: lumbar erector spinae muscle
M: male
M1: primary motor cortex
MCI: motor control impairment
MDCS: multidimensional classification system
MP: mixed pain
NcP: nociceptive pain
NpP: nocioplastic pain
NRS: numerical rating scale
ODQ: Oswestry low back pain Disability Questionnaire
ÖMPQ: Örebro Musculoskeletal Pain Screening Questionnaire
PASS‐20: Pain Anxiety Symptoms Scale
PCS: Pain Catastrophizing Scale
PES: peripheral electrical stimulation
PSEQ: Pain Self‐Efficacy Questionnaire
RMS: root mean square
SM: superficial fibres of the multifidus muscle
T9: ninth thoracic vertebrae
*SD*
: standard deviation
TMS: transcranial magnetic stimulation
TrA: transversus abdominis muscle

## 1 INTRODUCTION

Low back pain (LBP) is a leading global cause of disability (Hoy et al., 2014), affecting 60%–80% of people at some point in their lives. The prevalence and economic burden of LBP continue to rise (Cieza et al., 2020). Failure to consider the heterogeneity of LBP is likely to contribute (Falla & Hodges, 2017) to the modest effects (Froholdt et al., 2012) of recommended active physical interventions (Savigny et al., 2009). Success of interventions, such as those that aim to address how people with LBP move and function (Macedo et al., 2014), is likely to be improved if it is tailored to consider the underlying pain mechanisms.

The persistence of LBP may involve several mechanisms. For some, LBP may be maintained by a contribution from ongoing nociceptive input (i.e., nociceptive pain; NcP) from suboptimal tissue loading, which could be secondary to maladaptive responses of the movement system (e.g., excessive muscle guarding) (Hodges & Tucker, 2011). NcP is thought to be characterised by anatomically localised pain, consistently aggravated/relieved by specific postures and movements, and proportional to the initial trauma or injury (Shraim et al., 2020). For others, persistent LBP can be mediated by sensitisation mechanisms in the central nervous system involved in nociceptive pain processing or central sensitisation (Woolf, 2011), which is now referred to as nociplastic pain (NpP). In NpP, pain may persist in the absence of nociceptive input and is often linked to maladaptive cognitions about pain such as catastrophising and fear‐avoidance behaviour (Smart et al., 2012a).

Clinical features of pain presentation differ between individuals with LBP that is mediated by NcP and NpP mechanisms (Shraim et al., 2020). From the perspective of movement, specific kinematic such as, poor control of spine movements (e.g., dissociation of the thoraco‐lumbar spine) (Hemming et al., 2018; Sheeran et al., 2012), and muscle activation characteristics (e.g., asynchronous activation of erector spinae muscles) (Astfalck et al., 2010) differ between individuals with different NcP presentations. From the neural perspective, differences in functional connectivity and neuro‐immune signatures have been correlated with clinical presentations of NpP (Alshelh et al., 2021). Current clinical classifications combine assessment of maladaptive psychosocial features with posture/movement (O'Sullivan, 2005). None has yet combined consideration of motor control and detailed analysis of underlying pain mechanism.

Characteristics of M1 differ between individuals with and without persistent LBP, and to some extent, between participants (Elgueta‐Cancino et al., 2018; Schabrun et al., 2015; Tsao, Danneels, & Hodges, 2011a, 2011b). This variability might be related with individual‐specific responses to pain. Evidence in early stages of LBP shows that variability in corticospinal excitability and organisation of the primary motor cortex (M1) (e.g., volume of muscle representation) can predict pain persistence (Seminowicz et al., 2019). Further, features of M1 organisation (Tsao, Danneels, & Hodges, 2011b) relate to impairments of trunk muscles control (e.g., timing of muscle activation (Tsao et al., 2008)), and coordination of back movements (Elgueta‐Cancino et al., 2018) in persistent LBP. Although the relationship between M1 changes and clinical outcomes (e.g. pain persistence) remain unclear, the impact of experimental pain on M1 in motor learning paradigms has been describe as maladaptive plasticity (e.g., corsticospinal inhibition, poor sensory–motor integration) (Rohel et al., 2021), with negative impact on motor performance (e.g., poor retention of new motor skills) (Sanderson et al., 2021). It is plausible that the relationship between M1 changes and movement might depend on pain mechanism, as NpP is generally considered maladaptive pain processing (Shraim et al., 2020), whereas NcP is considered as a proportional response to nociceptive neuron activity (Shraim et al., 2020). The identification of specific characteristics of cortical organisation in subgroups of pain presentations could provide insight into underlying mechanisms of pain and movement dysfunction in LBP.

We aimed to explore whether features of M1 representation of the back muscles differ between individuals with different presumed pain mechanisms of LBP persistence and movement features. We hypothesised that features of M1 representations of the back muscles in individuals with predominant NcP would differ consistently from pain‐free individuals, but this may not be apparent when pain is maintained by NpP. M1 representation of the back muscles was investigated in four subgroups LBP; two groups with presumed NcP, and a distinct type of motor control impairment, one group with features consistent with NpP, and a mixed group with combined features of NcP and NpP.

## 2 METHODS

### 2.1 Study design

Cross‐sectional exploratory experimental study investigating cortical organisation in individuals with LBP with different presumed underlying pain mechanism; NcP, NpP and mixed pain pattern (MP).

### 2.2 Participants

**FIGURE 1:**
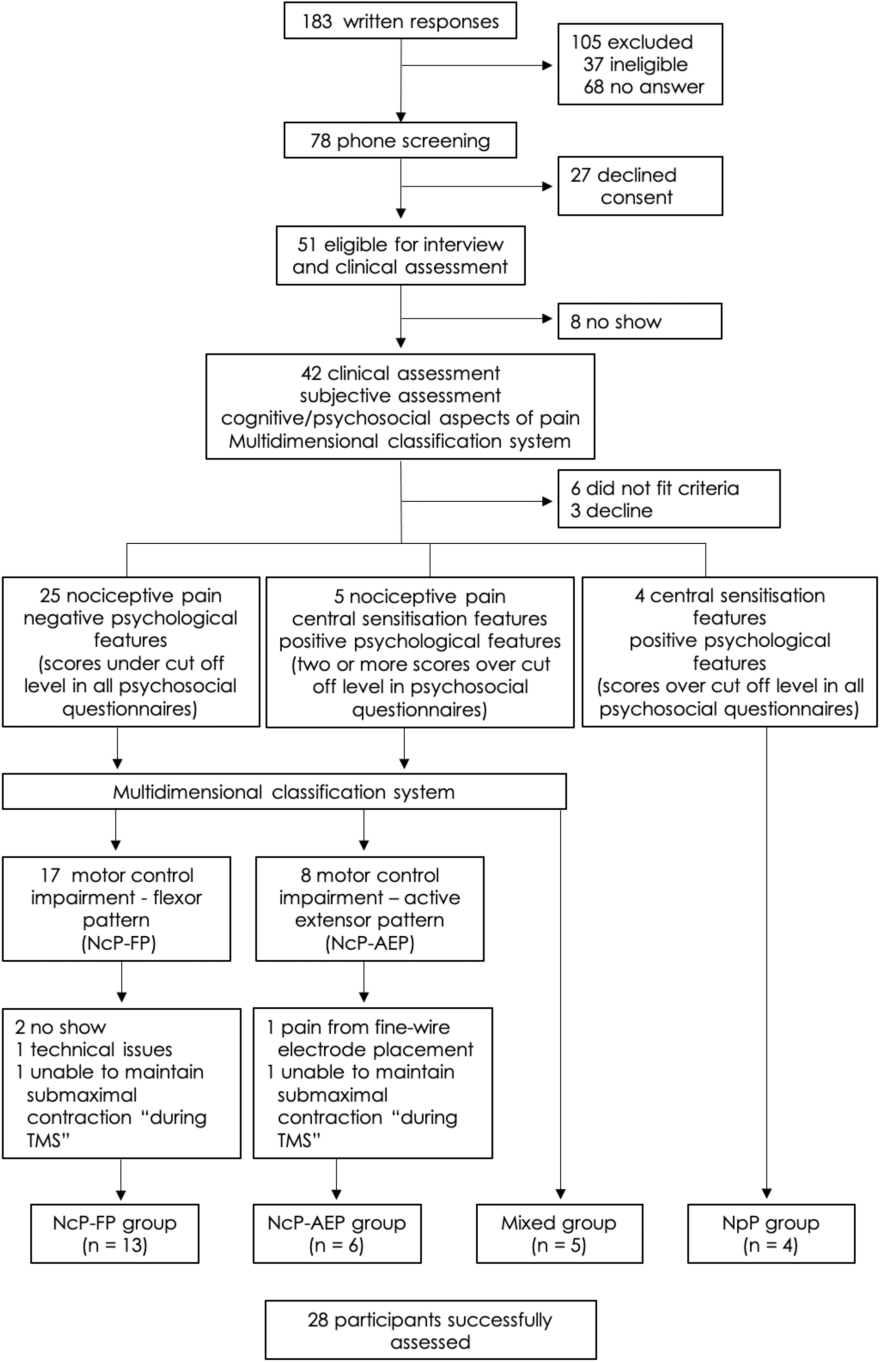
Recruitment and classification workflow. NcP‐AEP, nociceptive pain‐active extensor pattern group; NcP‐FP, nociceptive pain‐flexor pattern group; NpP, nociplastic pain group; TMS, transcranial magnetic stimulation

Participants were recruited from the university community, university musculoskeletal clinic and a commercial recruitment agency using online resources (university newsletter, webpages and social media) (Figure 1). Potential participants were eligible if they were aged between 18 and 65 years with LBP for >8 weeks that was located between the rib cage and buttocks (without leg pain) and an intensity of >4/10 on a numerical rating scale (NRS; no pain = 0, worst pain imaginable = 10) in the past 7 days. For this exploratory study, we intended to recruit participants with different presentations (see below) but acknowledged that the proportion of participants recruited with each pain type would differ based on the relative prevalence of specific pain presentations and the willingness of individuals with different presentations to volunteer for this invasive and intensive study. The sample size was based on a sample of convenience within these constraints and involved 28 participants.

Potential participants were screened using a questionnaire, telephone interview and safety questionnaire for use of transcranial magnetic stimulation (TMS) (Wassermann, 1998). Potential participants were excluded if they had the following: contraindications for TMS (stroke, metal in head, epilepsy and use of antidepressants); or participation in training that might change cortical representation of the trunk muscles (e.g., motor skill training programme for trunk muscles). Participants with suspected neuropathic pain (involving changes associated with damage or dysfunction of neural tissue; Treede et al., 2008) were also excluded. This was identified by evidence or history of nerve involvement (e.g., positive straight leg raise test, pain referral below the buttock line and/or altered sensation [numbness and/or pins and needles in one or both legs]). Additional criteria for inclusion of NcP are provided below. Participants provided written informed consent and the Institutional Medical Research Ethics Committee approved the study.

### 2.3 Pain mechanism classification

Participants with LBP were classified based on their clinical presentation of LBP according to the presumed underlying pain mechanisms, posture and movement profiles and cognitive/psychological features before the experimental procedures began (Figure 1). The likely underlying pain mechanism for LBP was determined based on detailed assessment of the characteristics of LBP following recommendations of clinically validated methods (Nijs et al., 2015; Shraim et al., 2020; Smart et al., 2012a, 2012b) (Table 1) and an in‐depth interview to explore the clinical indicators of NcP and NpP mechanisms (Smart et al., 2010, 2011; Smart et al., 2012a, 2012b). This included injury history, pain behaviour (24‐hour pattern, aggravating and pain easing factors), questions about lifestyle, daily habits (work status, exercise habits, leisure activities, family support, etc.). In addition, physical examination including functional and physiological movement assessment of the lumbar spine was conducted by an experienced physiotherapist (LS). Cognitive/psychosocial aspects of the patient's pain (beliefs, fears, stress/anxiety, mood, coping strategies and work‐/home‐related factors) were assessed with a series of standard questionnaires (Table 2). All questionnaires were administered in the same order to maintain consistency. Pre‐determined and validated cut‐off points of those questionnaires were used to identify presence of abnormal psychosocial features (Macedo et al., 2012). Participants with features of NcP pattern were further classified using multidimensional classification system (MDCS), a system with good validity and reliability (Dankaerts & O'Sullivan, 2011; Dankaerts, O'Sullivan, Straker, et al., 2006; Fersum et al., 2009), to evaluate features of their motor control impairment (MCI) type (Dankaerts, O'Sullivan, Burnett, & Straker, 2006a). Those with flexion pattern (FP) MCI and active extension pattern (AEP) MCI (Table 3) were included. These subgroups were selected for their different presentation of back muscle activity (Dankaerts, O'Sullivan, Burnett, & Straker, 2006a, 2006b), which we predicted would be associated with different changes motor cortex maps.

**TABLE 1:** Characteristics used to identify likely underlying mechanism for pain^a^

Criterion	Nociceptive pain	Nociplastic pain
History of pain	Clear history of pain evolution, usually related with specific events If onset was gradual—related to change in the level of activity, etc.	Unclear history, family history of ‘bad backs’ and or disabling diseases No clear injury or mechanism for their pain Disproportionate to injury mechanism
	Clear pattern of easing and pain provoking movement behaviours	No clear mechanical easing or provoking pattern
	Conceptions about the nature of their problem related with specific activities/level of fitness—weak muscles, muscle imbalance, stress, work, lack/too much physical activity, etc.	Pain could be related to change in activity, but also to personal events with emotional content.
	Moderate control of their symptoms—able to relate strategies to decrease pain and factors that aggravate it.	Feel unable to control symptoms, pain out of their control, usually rely on family members/others to help.
	Clear periods of symptoms and remission	
	Some previous benefit from physiotherapy or other movement related therapy	
Lifestyle	Variable, usually active despite pain, some avoidance of activities that provoke pain, etc.	Usually relies on family members/others to help
	Working/studying	Changed jobs due to LBP
	Physically/socially active	Unable to work or socialise due to their LBP
Area of pain	Confined to an anatomical region of the lower lumbar region, can be unilateral, central bilateral, swapping sides	Diffuse/variable area of symptoms
	Proportional pain response to palpation affected area	Disproportionate reaction to palpation of the affected area
	Proportional to injury mechanism and able to relate to specific activity or change in activity and proportional pain response to provocative movements	Unpredictable pattern of pain provocation
Aggravating postures/activities	Proportional to loading applied	Disproportionate and inconsistent to applied load
	Depending on the pattern: NP‐FP—flexion‐related activities, e.g., driving, prolonged sitting, prolonged cycling, lifting, bending, etc.	Unrelated to the mechanical pattern, hyperalgesia
	NP‐AEP—extension‐related activities, e.g., standing/walking/running long periods of time	
Easing postures/activities	Depending on the pattern: NP‐FP—extension postures/movement—standing, sitting with back support NP‐AEP—flexion postures/movements—crook lying, slumped sitting	Unclear relationship or non‐existent relationship
24‐h behaviour	Variable and usually related to level and type of activity	Always present, likely to interfere with daily activities

^a^ Adapted from Nijs et al. (2015) and Smart et al. (2010).

**TABLE 2:** Questionnaires used for classification^a^

Questionnaire	Domain	Description	Cut off points
Oswestry low back pain Disability Questionnaire (ODQ)	Disability	0–20 minimal disability, 21–40 moderate disability, 41–60 severe disability, 61–80 crippled, 81–100 bed bound	Severe disability score > 41%
Pain Anxiety Symptoms Scale (PASS‐20)	Psychological—fear and anxiety related to pain	0–100 point scale	Score >43
STarT Back Screening Tool	Physical and psychological	Risk for developing disabling LBP	Score >5
Örebro Musculoskeletal Pain Screening Questionnaire (ÖMPQ)	Psychological	24‐item score between 11 and 192	Score >103
Pain Catastrophizing Scale (PCS)	Psychological—catastrophisation	Total score 0–52 Three subscales—rumination, magnification and helplessness	Score >30
Pain Self‐Efficacy Questionnaire (PSEQ)	Psychological—self‐efficacy	10 items; 0–100 point scale	Score <41
Coping Strategies Questionnaire (CSQ)	Psychological—coping	Scale 0–36	Score <11

^a^ Adapted from Macedo et al. (2012).

**TABLE 3:** Multidimensional classification system (MDCS)

Flexion pattern features	Active extension pattern features
Loss of lumbar lordosis, tendency towards flexed lumbar spine	Active lumbar hyperlordosis, tendency towards actively adopting extended lumbar spine.
Pain provoked by movements/postures involving lumbar flexion (e.g., slouched sitting)	Pain provoked by movement/postures involving lumbar extension (e.g., hyperlordotic standing)
Pain eased by movements/postures involving lumbar extension (bending backwards)	Pain eased by movement/postures involving lumbar flexion (e.g., bending forwards)

Using these criteria, participants were classified into the following groups: (i) nociceptive pain group (NcP); with likely ongoing nociceptive contribution linked to flexion pattern (NcP‐FP, *n* = 13) or active extension pattern (NcP‐AEP, *n* = 6) MCI and psychosocial features within ‘normal’ limits; (ii) nociplastic pain group (NpP, *n* = 4), which included those with absence of mechanical pain behaviour and predominant features of central sensitisation (Smart et al., 2010, 2011; Smart et al., 2012a, 2012b) and psychosocial features above normal limits; and (iii) mixed pain group (MP) with features of both NcP and NpP that included psychosocial features outside normal limits (*n* = 5). Participants were classified before the experimental measures began.

### 2.4 Electromyography

Pairs of intramuscular fine‐wire electrodes (Teflon‐coated stainless steel wires, 75‐um diameter, 1 mm Teflon removed, tips bent back ~1 and ~2 mm to form the hooks) were used to record electromyography (EMG) from the back muscles on the most painful side or on the dominant side, when pain was symmetrical. Electrodes were inserted with guidance of real‐time ultrasound imaging at the level of L4/L5 lamina into the short, deep fibres of the multifidus muscle (DM), superficial fibres of multifidus (SM) and lumbar erector spinae (LES). Pairs of surface EMG electrodes (Ag/AgCl) were placed over the erector spinae muscle 2–3 cm lateral to T9 spinal process (TES). The ground electrode was positioned over the anterior superior iliac spine. EMG data were pre‐amplified 2000 times, band‐pass filtered (20–1000 Hz) and sampled at 2000 Hz using a Power 1401 Data Acquisition System with Signal2 software (Cambridge Electronic Design, CED, UK). Data were exported and analysed with Matlab 6.5 (The MathWorks, USA).

### 2.5 Motor cortex mapping

The representation of outputs to the back muscles at M1 was mapped with TMS using a figure‐of‐eight coil placed with the crossover position over the target scalp sites and the handle orientated posteriorly along the sagittal plane as described in detail elsewhere (Tsao, Danneels, & Hodges, 2011b). As 120% of motor threshold of the back muscles exceeds the maximum stimulator output (Tsao et al., 2010; Tsao, Tucker, & Hodges, 2011), stimulation was set to 100% for all participants. Stimuli were delivered at 40 points on a grid with 5 × 8 stimulation sites separated by 1 cm over each hemisphere (medio‐lateral dimension: midline to 4‐cm lateral; antero‐posterior dimension: between 5‐cm anterior to the vertex and 2‐cm posterior to this point). TMS mapping was guided using a Brainsight 2™ navigation system (Rogue Research, Canada) based on reference points obtained from the location of the vertex and using an international 10/20 electrode placement system (Jasper, 1958). Participants performed three maximum voluntary contractions (MVC) of the back muscles (~3‐s duration). With the participant sitting upright in a chair with a backrest at 90°, arms crossed and feet on the floor, they performed isometric back extension against manual resistance applied to the upper thorax. The maximum root mean square (RMS) amplitude across the trials for DM was identified. Five TMS pulses were delivered at each point on the grid (inter‐stimulus interval: ~5 s) while participants sustained a contraction of the back muscles by leaning forward with a straight back to match 10% of the MVC RMS EMG recorded for DM. Visual feedback was provided and monitored by the experimenter. Verbal feedback was provided to make adjustments to EMG amplitude if necessary. Procedures adhered to the TMS checklist for methodological quality (Chipchase et al., 2012).

## 3 DATA ANALYSIS

### 3.1 Motor cortex map analysis

The onset and offset of the motor evoked potential (MEP) were visually determined from each individual stimulation, and the RMS EMG of each MEP was calculated between these points. The background RMS EMG, calculated between 500 and 5 ms before the TMS pulse, was subtracted from the corresponding MEP. For each stimulation site, the MEP onset, offset, and amplitude (i.e., RMS EMG) were averaged across responses. A topographical map of MEP amplitudes was generated, corresponding to the respective scalp sites. The amplitude of this MEP map was normalised to the highest amplitude (hot spot), and values smaller than 25% of the peak were removed (Tsao et al., 2008).

The *centre of gravity* (CoG), or weighted amplitude, of the map is indicative of the location of the map in M1. Previous research has shown that changes in CoG location represent reorganisation of M1 (Rossini et al., 2003; Thickbroom Gary et al., 2003). CoG was calculated for medial–lateral (*x*‐coordinate) and anterior–posterior (*y*‐coordinate) directions using the formulas: CoG_*x*_ = ∑*z*_*i*_ *x*_*i*_/∑*z*_*i*_; CoG_*y*_ = ∑*z*_*i*_ *y*_*i*_/∑*z*_*i*_, where *x*_*i*_ and *y*_*i*_ refer to individual scalp sites and *z*_*i*_ is the corresponding (normalised) MEP amplitude (Tsao et al., 2008). Inter‐subject variability of the location of M1 maps was quantified by subtracting the location of individual CoGs relative to the group mean CoG location (referred to as *CoG variation*). This measure represents the heterogeneity of M1 organisation between participants with similar clinical presentation of pain. It was calculated separately in the medial–lateral (*x*) and anterior–posterior (*y*) directions and as a vector.

*Map volume* is a measure of the total excitability of the cortical representation (Brasil‐Neto et al., 1992; Wassermann et al., 2008; Wilson et al., 1993); it was calculated as the sum of normalised MEPs recorded across all scalp sites. The *map area* of representation was determined as the total number of grid sites that evoked an MEP with an amplitude >25% of the MEP at the hot spot. The *overlap* between pairs of representation maps is an indicator of the discrete location of representations of different muscles (Dechent & Frahm, 2003; Devanne et al., 2002). Overlap of representations of separate muscles, as revealed by TMS mapping, has identified: overlap for muscles that require coordinated activity for specific tasks (Devanne et al., 2006; Marconi et al., 2007) and greater overlap for specific muscle representations in chronic pain conditions (Schabrun et al., 2015; Te et al., 2017; Tsao, Danneels, & Hodges, 2011b). It was quantified for each combination of muscle pairs as the number of sites that include an MEP in both muscles, expressed as a proportion of the total number of active sites summed across both muscles, minus the area of overlap (Massé‐Alarie et al., 2017). Overlap was calculated for: DM/SM, DM/LES, DM/TES, SM/LES, SM/TES, and LES/TES.

### 3.2 Statistical analysis

Normality of distribution of all variables was tested with Shapiro–Wilk's test. Parameters of M1 map (CoG location, CoG variation, map volume, area and overlap) were compared between groups (NcP‐FP, NcP‐AEP, MP, and NpP) using one‐way ANOVA. Welch test was applied when unequal variances were found. Pair‐wise post hoc comparisons were performed using Duncan's multiple range test or Games‐Howell test if the variances were unequally distributed. Spearman's rank was used to investigate the relationship between pain intensity and map parameters for each group. Significance was set at α < 0.05. Effect sizes were calculated using eta squared, which measures the proportion of variance associated with or accounted for by each of the main effects. Consistent with recommendation for exploratory studies (Perneger, 1998), no adjustment was made for multiple comparisons. Unless otherwise indicated, data are presented as mean and standard deviation (mean [*SD*]) throughout the text, figures and tables.

## 4 RESULTS

### 4.1 Sample characteristics

One hundred and eighty‐three individuals responded to advertisements. After screening and assessment (Figure 1), twenty‐eight volunteers were recruited and completed the study. Table 4 presents sample demographics.

**TABLE 4:** Participant demographics

	NcP‐FP	NcP‐AEP	MP	NpP
	*n* = 13	*n* = 6	*n* = 5	*n* = 4
	Mean (*SD*)	Mean (*SD*)	Mean (*SD*)	Mean (*SD*)
Age (years)	32 (10)	41 (12)	38 (6)	>30 (5)
Sex (female/male)	4/9	5/1	3/1	>3/2
Weight (kg)	73.5 (14.4)	76.5 (9.7)	71.3 (4.4)	>59.6 (8.3)
Height (cm)	175.5 (9.7)	164.5 (11.0)	168 (4.7)	>162.8 (5.0)
Pain intensity (NRS)	4.2 (1.7)	5.5 (2.7)	5.0 (2.2)	>5.4 (1.7)
OQD	16.5 (8.5)	20.3 (8.5)	30.4 (10.3)	>40.5 (11)
STarT Back	2.5 (1.3)	2.7 (1.4)	4 (1.4)	>7 (0.8)
ÖMPQ	73.2 (17.0)	78.2 (11.4)	110.6 (22.9)	>140.8 (34.0)
PASS‐20	24.5 (13.4)	22.8 (11.1)	42.2 (12.9)	>57.5 (17.7)
PCS	16 (12.9)	20.8 (7.7)	41.4 (11.9)	>32.5 (4.8)
PSEQ	50.8 (6.6)	48.2 (6.4)	34.4 (11.3)	>30.8 (8.1)
CSQ	30.8 (11.4)	36.8 (14.1)	53.6 (20.9)	38.8 (11.1)

Abbreviations: CSQ, Coping Strategies Questionnaire; NcP‐FP, nociceptive pain‐flexion pattern group; NcP‐AEP, nociceptive pain‐active extension pattern group; NpP, nociplastic pain group; MP, mixed pain group; NRS, numeric rating scale; ODQ, Oswestry low back pain Disability Questionnaire; PASS‐20, Pain Anxiety Symptoms Scale; ÖMPQ, Örebro Musculoskeletal Pain Screening Questionnaire; PCS, Pain Catastrophizing Scale; PSEQ, Pain Self‐Efficacy Questionnaire.

DM MEPs were successfully recorded from all participants. Despite high‐quality EMG recordings, no MEPs could be elicited from the SM muscle of one NcP‐FP participant, LES of one NcP‐FP and one NpP participant, or TES of one NcP‐AEP participant. Data for an individual EMG channel were rejected in eight participants due to poor quality of recordings. These included EMG signals of SM in one NcP‐AEP participant; LES in two NcP‐FP participants and four NcP‐AEP participants; and TES in one NcP‐FP participant.

### 4.2 Comparison of M1 organisation between groups

**FIGURE 2:**
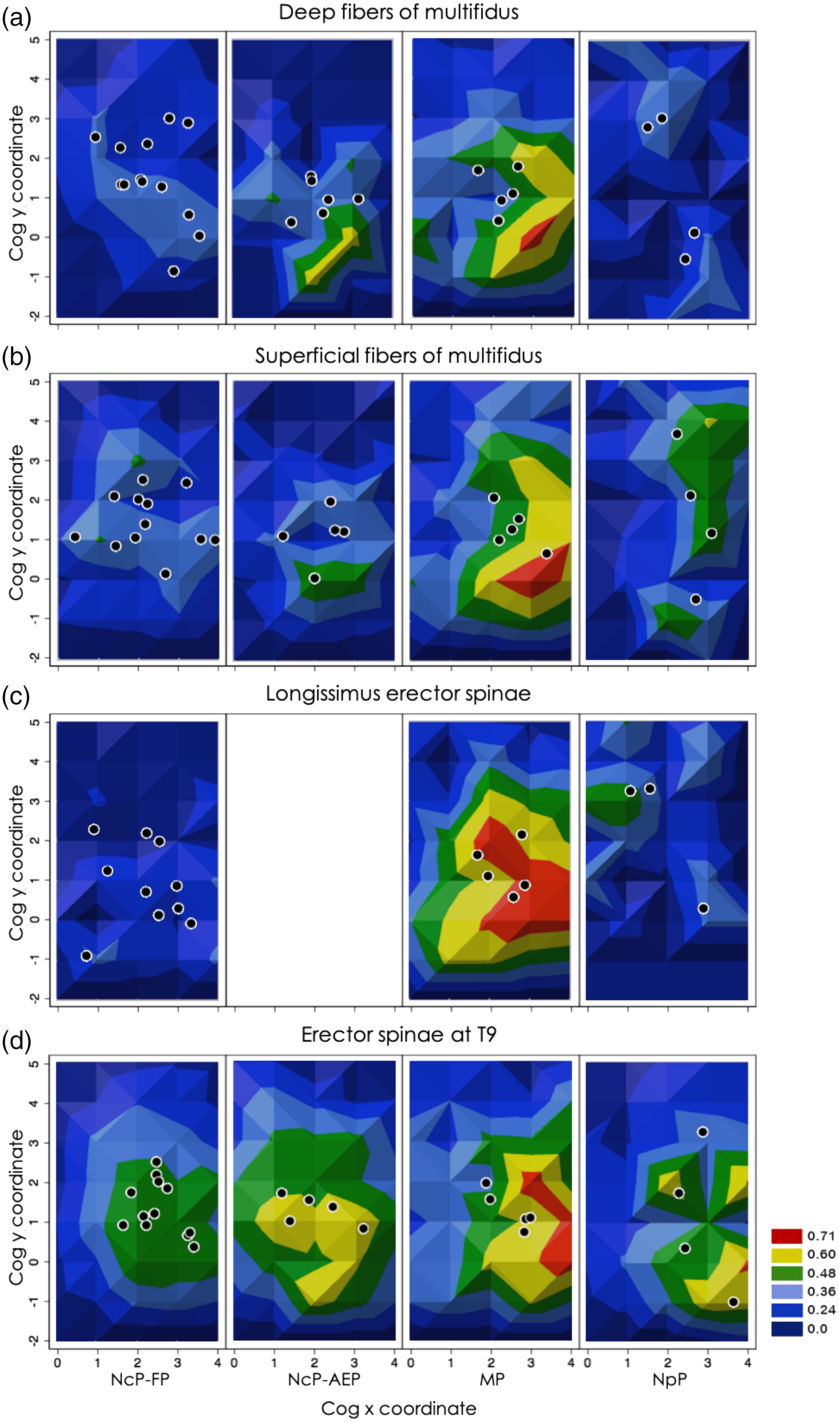
Averaged M1 representations of each muscle and individual CoGs, by CLBP subgroup: nociceptive pain‐flexor pattern group (NcP‐FP), nociceptive pain‐active extensor pattern group (NcP‐AEP), mixed pain group (MP) and central sensitisation pain group (nociplastic pain group [NpP]). Maps are shown for (a) deep fibres of multifidus, (b) superficial fibres of multifidus, (c) lumbar erector spinae and (d) thoracic erector spinae. Closed circles represent location of centre of gravity for each participant in each muscle. As maps are generated from average of individual maps that were normalised to peak, averaged maps with large peaks indicate that the location of the highest peak was similar for all individuals; flatter maps indicate high variation of peak location between individuals. No data are available for lumbar erector spinae for the NcP‐AEP group because of poor quality of EMG recordings for four participants

Figure 2 presents the averaged map representation of DM, SM, LES and TES at M1, for each group. There were no differences in the location of the CoG between groups for any muscle in anterior–posterior or medio‐lateral directions (Table 5). No difference was found between groups in the percentage of overlapped areas of DM/SM or SM/TES (Table 5). For the overlap between DM/LES, this was greater for the MP than the NcP‐FP (main effect group: *F*_[1, 15]_ = 8.1, *p* = .004, *η* ^2^ = .522; post‐hoc *p* = .001) and NpP groups (*p* = .02). DM/TES overlap was larger in the MP than NcP‐FP (*p* = .008) and NpP (*p* = .02) groups, but not different from the NcP‐AEP group (*p* = .07). Overlap of SM/LES was greater for the MP than NcP‐FP (main effect group: *F*_[2, 14]_ = 4.3, *p* = .03, *η* ^2^ = .403, post hoc *p* = .02) and NpP (*p* = .04) groups; and LES/TES overlap was greater for the MP than NcP‐FP (main effect group: *F*_[2,14]_ = 7.4, *p* = .007, *η* ^2^ = .513; post hoc *p* = .004) and NpP (*p* = .02) groups.

**TABLE 5:** Between‐group comparisons of motor cortex organisation

	NcP‐FP		NcP‐AEP		MP		NpP		Group effect		
Variable	Mean (*SD*)	*n*	Mean (*SD*)	*n*	Mean (*SD*)	*n*	Mean (*SD*)	*n*	*F*	*p*	*η* ^2^
Map volume DM	5.43 (3.31)	13	5.39 (2.81)	6	10.34 (5.91)	5	5.52 (2.8)	4	2.37	0.096	0.228
Map volume SM	5.96 (4.34)	12	6.46 (3.36)	5	11.41 (7.52)	5	7.42 (4.48)	4	1.49	0.244	0.169
Map volume LES	4.30 (3.01)	10	^a^		10.62 (2.84)	5	5.96 (3.46)	3	7.27	**0.006**	0.492
Map volume TES	8.75 (4.87)	12	12.84 (3.47)	5	11.55 (5.87)	5	9.19 (4.54)	4	1.06	0.388	0.126
CoG vector DM	3.01 (0.69)	13	2.39 (0.57)	6	2.60 (0.39)	5	2.97 (0.46)	4	1.74	0.186	0.178
CoG vector SM	2.81 (0.89)	12	2.50 (0.65)	5	2.94 (0.38)	5	3.42 (0.64)	4	1.19	0.336	0.140
CoG vector LES	2.60 (0.71)	10	^a^		2.73 (0.52)	5	3.32 (0.39)	3	1.54	0.247	0.170
CoG vector TES	2.96 (0.53)	12	2.48 (0.63)	5	2.89 (0.26)	5	3.37 (0.87)	4	1.81	0.175	0.200
CoG DM *x*‐coordinates	2.35 (0.79)	13	2.15 (0.56)	6	2.27 (0.39)	5	2.12 (0.53)	4	0.19	0.901	0.023
CoG SM *x*‐coordinates	2.27 (0.98)	12	2.17 (0.60)	5	2.58 (0.52)	5	2.66 (0.36)	4	0.47	0.706	0.060
CoG LES *x*‐coordinates	2.17 (0.92)	10	^a^		2.35 (0.53)	5	1.84 (0.95)	3	0.36	0.706	0.045
CoG ES T9 *x*‐coordinates	2.54 (0.56)	12	2.03 (0.83)	5	2.51 (0.54)	5	2.81 (0.61)	4	1.30	0.300	0.150
CoG DM y‐coordinates	1.50 (1.13)	13	0.97 (0.45)	6	1.17 (0.56)	5	1.33 (1.82)	4	0.65	0.6033	0.044
CoG SM y‐coordinates	1.44 (0.73)	12	1.09 (0.70)		1.29 (0.53)	5	1.6 (1.75)	4	0.29	0.835	0.038
CoG LES y‐coordinates	0.85 (1.07)	10	^a^		1.26 (0.63)	5	2.28 (1.73)	3	1.98	0.173	0.210
CoG TES y‐coordinates	1.35 (0.69)	12	1.30 (0.37)	5	1.29 (0.49)	5	1.07 (1.85)	4	0.04	0.958	0.014
Variation of CoG vector DM	1.15 (0.68)	13	0.60 (0.31)	6	0.56 (0.270	5	1.63 (0.24)	4	15.18	**0.0003**	0.369
Variation of CoG vector SM	0.79 (0.33)	12	0.73 (0.40)	5	0.62 (0.19)	5	1.53 (0.81)	4	3.01	0.052	0.202
Variation of CoG vector LES	1.21 (0.60)	10	^a^		0.71 (0.190)	5	1.52 (0.64)	3	2.47	0.118	0.365
Variation of CoG vector TES	0.79 (0.33)	12	0.73 (0.40)	5	0.62 (0.19)	5	1.53 (0.81)	4	4.21	**0.017**	0.248
Variation DM *x*‐coordinates	0.66 (0.40)	13	0.40 (0.35)	6	0.27 (0.23)	5	0.44 (0.17)	4	1.88	0.160	0.190
Variation SM *x*‐coordinates	0.72 (0.62)	12	0.46 (0.32)	5	0.37 (0.31)	5	0.25 (0.21)	4	1.31	0.297	0.151
Variation LES *x*‐coordinates	0.73 (0.50)	10	^a^		0.45 (0.17)	5	0.7 (0.39)	3	0.78	0.478	0.094
Variation TES *x*‐coordinates	0.43 (0.34)	12	0.65 (0.40)	5	0.47 (0.13)	5	0.45 (0.32)	4	0.62	0.609	0.078
Variation DM *y*‐coordinates	0.85 (0.70)	13	0.34 (0.26)	6	0.44 (0.27)	5	1.56 (0.29)	4	15.31	**0.0004**	0.383
Variation SM *y*‐coordinates	0.61 (0.35)	12	0.44 (0.49)	5	0.39 (0.30)	5	1.28 (0.93)	4	3.01	0.052	0.291
Variation LES *y*‐coordinates	0.85 (0.58)	10	^a^		0.50 (0.29)	5	1.33 (0.58)	3	2.44	0.121	0.245
Variation TES *y*‐coordinates	0.59 (0.30)	12	0.30 (0.15)	5	0.39 (0.22)	5	1.43 (0.84)	4	3.68	0.059	0.500
Area DM	13.31 (6.93)	13	12.83 (6.82)	6	24.60 (7.370	5	10.5 (4.73)	4	4.43	**0.013**	0.357
Area SM	14.08 (8.11)	12	14.60 (8.76)	5	23.20 (7.60)	5	14.25 (8.85)	4	1.59	0.219	0.179
Area LES	10.90 (6.59)	10	^a^		24.80 (5.31)	5	10.33 (9.29)	3	7.89	**0.005**	0.513
Area TES	21.17 (8.80)	12	24.60 (5.46)	5	24.60 (8.56)	5	18.25 (10.72)	4	0.60	0.620	0.076
Overlap DM/SM	0.44 (0.24)	12	0.34 (0.21)	5	0.65 (0.20)	5	0.33 (0.2)	4	2.10	0.129	0.223
Overlap DM/TES	0.44 (0.22)	12	0.46 (0.18)	5	0.72 (0.15)	5	0.39 (0.16)	4	3.06	**0.050**	0.294
Overlap SM/TES	0.46 (0.24)	11	0.59 (0.12)	4	0.66 (0.18)	5	0.57 (0.17)	4	1.28	0.308	0.161
Overlap DM/LES	0.33 (0.22)	10	^a^		0.74 (0.12)	5	0.4 (0.11)	3	8.19	**0.004**	0.522
Overlap SM/LES	0.38 (0.28)	9	^a^		0.75 (0.11)	5	0.33 (0.2)	3	4.73	**0.027**	0.403
Overlap LES/TES	0.33 (0.23)	9	^a^		0.74 (0.13)	5	0.31 (0.2)	3	7.36	**0.007**	0.513

*Note:* Significant differences highlighted in bold.

Abbreviations: CoG, centre of gravity; LES, lumbar erector spinae; NcP‐FP, nociceptive pain‐flexor pattern; NcP‐AEP, nociceptive pain‐active extensor pattern; MP, mixed pain; NpP, nociplastic pain; DM, deep fibres of multifidus muscle; SM, superficial fibres of multifidus muscle; TES, erector spinae at level of T9.

^a^ Missing data of LES recordings in the NcP‐AEP group—this group was not included on the analyses because of the insufficient amount of data obtained (poor quality of electromyography recordings determined that maps were only obtained for two out of six participants of the group).

The LES map volume was greater in the MP than NcP‐FP (main effect group: *F*_[2, 15]_ = 7.3, *p* = .006, *η* ^2^ = .492, post hoc *p* = .002) and NpP (*p* = .03) groups, whereas the map volumes of the other muscles were similar across all groups, 23% of the variance can be accounted by the group differences of DM volume. Map area of DM was greater for participants in the MP than all other groups (main effect group: *F*_[3, 24]_ = 4.4, *p* = .01, *η* ^2^ = .357, post hoc: NcP‐FP *p* = .004; NcP‐AEP *p* = .01; NpP *p* = .008). Similarly, LES area was significantly larger for MP than NcP‐FP (main effect group: *F*_[2, 15]_ = 7.9, *p* = .005, *η* ^2^ = .513, post‐hoc *p* = .002) and NpP (*p* = .01) groups and accounted for 51% of the variance. Group effect accounted 22% of the variance of TES maps. There was no difference between groups in the map area of SM.

### 4.3 Comparison of M1 inter‐subject variation between groups

**FIGURE 3:**
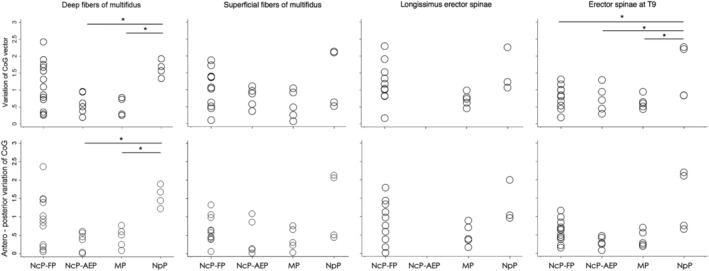
Variation in the location of individual centre of gravity (CoG) related to the mean by muscle and group. Data are shown for the vector (top panel) and *y*‐coordinates (bottom panel). NcP‐FP, nociceptive pain‐flexor pattern; NcP‐AEP, nociceptive pain‐active extensor pattern; MP, mixed pain; NpP, nociplastic pain

Average maps generated from individual maps for DM, SM, LES and TES muscles in M1 revealed differences in inter‐subject variation between groups (Figure 3). Greater CoG variability in DM map was measured in the NpP than MP (main effect group: *F*_[3, 10.9]_ = 15.2, *p* = .001, *η* ^2^ = .369, post‐hoc *p* = .002) and NcP‐AEP (*p* = .002) groups this accounting for a 37% of the variance. In addition, differences between groups in antero‐posterior variation of DM CoG location accounted by a 38% of the variance. The variation was greater for NpP than NcP‐AEP (main effect group: *F*_[3, 10.5]_ = 15.3, *p* = .001, *η* ^2^ = .383, post hoc *p* = .002) and MP (*p* = .004) groups, and narrowly missed significance compared with the NcP‐FP group (*p* = .051). There were no differences in CoG location in the medio‐lateral direction between groups. The CoG vector variation of SM was similar for all groups. Although SM showed a similar trend to that for DM distribution of CoG variability for NpP group in the anterior–posterior direction and group effects was accounted for 29% of the variance, the test narrowly missed significance (main effect group: *F*_[3, 22]_ = 3.0, *p* = .05, *η* ^2^ = .291). The CoG variation of LES did not differ between groups (main effect group: *F*_[3, 22]_ = 4.2, *p* = .118, *η* ^2^ = .365). The CoG vector variation for TES was greater for the NpP than all other groups (main effect group: *F*_[3, 22]_ = 4.2, *p* = .02, *η* ^2^ = .245): NcP‐FP (post‐hoc *p* = .006), NcP‐AEP (*p* = .01) and MP (*p* = .006). Variation of CoG location of TES between groups accounted for 50% of the variance and in the antero‐posterior direction were close to significance (main effect group: *F*_[3, 22]_ = 3.7, *p* = .06, *η* ^2^ = .500), but not in the medio‐lateral direction (main effect group: *F*_[3, 25]_ = 0.62, *p* = .61, *η* ^2^ = .078).

### 4.4 Relationship between M1 organisation of back muscles and pain

In the NcP‐FP group, DM/SM overlap was significantly correlated with pain scores (rho = .58, *p* = .05), that is, individuals with larger areas of overlap reported greater pain. There was a similar relationship for DM/LES overlap (rho = .78, *p* = .007). The individual variation in location of CoG of SM in antero‐posterior direction correlated with pain (rho = .63, *p* = .02). For the NcP‐AEP group, coordinates in medio‐lateral direction and antero‐posterior direction of CoG variation of SM were correlated with pain (*x*‐coordinate: rho = .9, *p* = .04, *y*‐coordinate: rho = −.9, *p* = .04), that is, individuals with larger SM CoG variation in medio‐lateral direction and those with less variation in antero‐posterior direction, reported greater pain. In the MP group, participants with smaller map volume of SM also have higher levels of pain (rho = −.97, *p* = .005), and CoG of LES was located more posterior in individuals with higher pain (rho = −.97, *p* = .005). Greater variation in the location of CoG in antero‐posterior direction of TES correlated with worse pain (*y*‐coordinate: rho = .97, *p* = .005). NpP group showed no correlation between any of TMS map variables and intensity of pain (all *p* > .6).

## 5 DISCUSSION

This study provides preliminary evidence of differences in the M1 organisation between individuals with persistent LBP who are classified using clinical criteria into groups with different presumed pain mechanisms. Key observations were that the NpP group had greater variation in CoG location than MP and NcP groups, the MP group had greater overlap between representations of separate muscles than NcP and NpP groups, and there was no difference between individuals with NcP with different MCI patterns (flexion or active extension). Taken together, these data suggest that M1 organisation of back muscles depends on the predominant pain mechanism. These findings provide new insight into possible factors underlying changes in M1 organisation in LBP with possible implications for rehabilitation.

### 5.1 Differences in variability of M1 organisation between groups

Difference in variation between groups has several possible interpretations. First, greater inter‐individual variation of M1 map location (CoG) in NpP appears consistent with variation in motor strategies adopted by people with some pain presentations (Dankaerts et al., 2009). Several studies have identified a relationship between specific motor features and variation in cortical mechanisms. For instance, the amplitude of delay in activation of TrA is related to the amplitude of shift of location of M1 CoG in individual with LBP relative to controls (Tsao et al., 2008); variation in the ability to voluntarily activate the multifidus muscle correlates with the reduction of short interval intracortical inhibition and active motor threshold (Massé‐Alarie et al., 2015), and individuals with pain provoked in flexion show a relationship between proprioception and poor precision of movement control (Tong et al., 2017). Although, pain interference can induce changes in motor coordination of trunk muscles (Moseley & Hodges, 2005) how pain interference differs between pain mechanisms is not yet clear. None of these observations has been considered with respect to pain mechanism.

Second, differences in variation might relate to use (or training), which can change motor representations. Training of deep abdominal muscles shifts the M1 map CoG anteromedially (Tsao et al., 2010). Further, variation in the medio‐lateral CoG location of upper limb muscles correlates with strength and coordination patterns between synergistic muscles (Plow et al., 2014). It is plausible that the more homogeneous CoG location in the NcP groups might relate to a consistent relationship between movement and pain, including consistent activation of specific muscles to avoid pain. Greater variation in CoG location in NpP group might be expected as for these individuals pain is unrelated to specific movements. Thus, patterns of motor adaptation are likely to be highly variable with different strategies adopted by different individuals, as proposed by Hodges and Tucker (2011). There is evidence that ‘strength’ of connectivity between cortex and periphery differs with pain ‘type’. For instance, chronic pelvic pain is associated with strengthened connectivity of the corticospinal tract from the M1 representation of the pelvic floor muscles (Kutch et al., 2015). This concurs with overactivity of pelvic floor muscles as a possible nociceptive input (Asavasopon et al., 2014). In contrast, individuals with NpP experienced as visceral pain have less connectivity between M1 and pelvic floor when compared with people with chronic pelvic pain (Huang et al., 2016). These data support the notion that enhanced corticospinal connectivity and focal M1 representation may be expected in NcP, whereas NpP would be associated with more variable M1 representation. An underlying tenet of this hypothesis is that individuals with NpP would have less consistent/constrained corticospinal drive to paraspinal muscles.

Third, although, our results were underpowered to show a clear relationship, greater variability of M1 in NpP group concurred with the presence of abnormal psychosocial features. This could relate to fear of movement and anxiety that is commonly a feature of NpP (Smart et al., 2012a). This could underpin movement avoidance behaviours and changes in motor control (Karayannis et al., 2013). Fear to the movement has been shown to be associated with alterations in motor control features such as muscle activation (Karayannis et al., 2013) and poor endurance of lumbar extensor muscles (Larivière et al., 2010; Mannion et al., 2011). Such negative psychological factors and pain can interfere with task performance secondary to competition of cognitive resources (Mazaheri et al., 2014). Together, these mechanisms could increase movement variability and less consistent M1 output to back muscles.

Fourth, centrally mediated alterations in sensory input might increase variability in motor system organisation in those with LBP maintained by NpP. Changes in sensory sensitivity related to central sensitisation (e.g., reduced pressure pain threshold, Giesecke et al., 2004, and deep tissue hyperalgesia, O'Neill et al., 2007) may increase ‘noise’ in the central nervous system and impact motor performance via interference with the motor output (Faisal et al., 2008).

Fifth, it is possible that high variability in M1 organisation is explained by differences in brain anatomy (Ahdab et al., 2013). Previous research has shown moderate evidence that musculoskeletal conditions involving NpP (e.g., chronic pelvic pain and fibromyalgia) show morphological alterations (e.g., grey and white matter volume) in somatosensory, affective‐motivational and cognitive processing of pain regions (Coppieters et al., 2016). These changes might have methodological consequences for interpretation of TMS (e.g., TMS coil orientation relative to neurons, Inuggi et al., 2010). This could explain the greater variability of M1 organisation for the NpP group if this condition is consistently associated with changes in brain anatomy.

Regardless of the underlying mechanisms, greater variability of M1 organisation observed in those with a presumed NpP contribution to LBP has potential important implications for interpretation of adaptations to pain and warrants further investigation.

### 5.2 Greater overlap of M1 representations in MP group

All groups showed overlap of M1 maps for superficial and deep muscles. This concurs with data showing greater overlap of M1 maps of deep and superficial back muscles in individuals with than without chronic LBP (Tsao, Danneels, & Hodges, 2011b). Additionally, our results described a group effect that accounted for 22% to 52% of differences, where the overlap was greatest for the MP group. Overlap of M1 representations is thought to aid coordination of synergist muscle control (Massé‐Alarie et al., 2017), but compromises independent muscle control (Schabrun et al., 2014). In the context of LBP, the overlap might be interpreted as negative/problematic for function, with less potential for discrete control or loss of independent fine‐tuning of these muscles (Tsao, Danneels, & Hodges, 2011b). This concurs with data that show individuals with greater overlap have poorer ability to separately move the thoracic and lumbar spine (Elgueta‐Cancino et al., 2018), which is a feature of individuals with LBP associated with motor control impairments (Hemming et al., 2018; Sheeran et al., 2012).

Although the MP group showed similar pain provocation movement patterns to the NcP‐FP group, their M1 overlap between superficial (LES and TES) and deep (DM) back muscles areas was greater than in the NcP and NpP groups. MP differed from the NcP group in that they presented with psychological features that were similar to the NpP group. Strong negative cognition about pain might underlie greater consistency of response (unlike the variation observed in NpP), but a more generalised protective co‐activation response (unlike the discrete movement features of NcP‐FP group). Previous data have shown a relationship between fear/anxiety of movement and protective responses to pain such as increased trunk stiffness (Karayannis et al., 2013) and reduced flexion–relaxation (Geisser et al., 2004). Based on these results, we hypothesise that the greater overlap of individual muscle representations might be explained by a consistent pattern of protection that is enhanced by fear.

### 5.3 Similar M1 organisation in the NcP group with different MCI types

The LBP groups with a probable ongoing nociceptive input were grouped based on movement features. The NcP‐FP group are characterised by a flat lumbar lordosis associated with less activation of lumbar ES than the NcP‐AEP group (Dankaerts et al., 2009). The NcP‐AEP group are characterised by a hyperextended spine and greater activation of SM and LES muscles (Dankaerts et al., 2009). These pain‐related differences in motor behaviour might be expected to be reflected in different M1 representations of back muscles, yet this was not observed. This concurs with recent data that have failed to observe systematic differences in LES EMG between FP and AEP groups (Sheeran et al., 2012). Alternatively, the number of participants in the NcP‐AEP group may have provided insufficient statistical power to observe differences particularly the absence of LES data, which showed an important group effect. As well as for the variability in the map representation of the DM, where the group effect explained more than a third of the change and despite the clear difference with NcP‐FP, this did not reach significance.

### 5.4 Clinical implications

These results provide foundation to consider that treatments for LBP may need to be tailored to individuals with contribution of different pain mechanisms to their symptoms. First, substantial changes in M1 organisation were observed in those with combined impairment of motor control and negative psychosocial conceptions. This implies interventions may need to address both muscle activation and the psychological features to address the changes. Second, irrespective of the mechanisms, all groups demonstrated some features of M1 organisation that differ from the data presented previously for healthy pain‐free individuals. Features of movement control might need to be considered in each group, but with different goals and methods.

### 5.5 Limitations

These results should be interpreted with consideration of several methodological limitations. First, the high inter‐subject variation and small sample size limited our capacity to identify relationships. Second, given the unequal gender distribution between groups, we cannot exclude that some observations might be explained by gender differences rather than the presumed pain mechanisms. Interpretation is complicated by the observation that sex hormones are potential modulators of brain excitability, but with effects that differ between women (Fillingim et al., 2009). Third, skilled activities such as sports (Tyc et al., 2005) or playing a musical instrument (Pascual‐Leone et al., 1995) are known to influence organisation of the M1. Although our participants did not declare any involvement of in these activities, other factors cannot be excluded. Finally, the current research did not include a control group and instead related the observations to the cited work that included comparison between individuals with unclassified LBP and control participants.

## 6 CONCLUSION

This study provides preliminary evidence of differences in M1 organisation of the back muscles in subgroups of LBP classified by presumed underlying pain mechanisms. Despite the small sample size, all groups showed some features of cortical organisation that differ from those reported in pain‐free individuals, but with differences between subgroups. Notably, NpP was characterised by greater inter‐subject variability of M1 organisation, which appears congruent with the lesser relevance of the interaction between movement/postures and pain. Further, the combination of motor and psychological variables related to pain in the MP group could explain its unique M1 features. Different treatments might be required to promote recovery for subgroups of LBP.

## CONFLICT OF INTEREST

Authors have no conflicts of interest to declare.

## AUTHOR CONTRIBUTIONS

E. E. C., L. S. and P. H. conceived and designed research; E. E. C., L. S., L. H. and P. H. performed experiments; E. E. C., S. S. and P. H. analysed data; E. E. C., L. S and P. H. interpreted results of experiments; E. E. C., L. S. and P. H. drafted manuscript; E. E. C., L. S., L. H., S. S. and P. H. edited, revised and approved the final version of manuscript.

### PEER REVIEW

The peer review history for this article is available at https://publons.com/publon/10.1111/ejn.15511.

## Data Availability

The datasets generated during and/or analysed during the current study are available from the corresponding author on reasonable request.
